# Development of a noninvasive redox imaging method that can stably detect radiation-induced intestinal injury

**DOI:** 10.1186/s11671-025-04355-y

**Published:** 2025-09-24

**Authors:** Kosei Adachi, Fuminori Hyodo, Abdelazim Elsayed Elhelaly, Koki Ichihashi, Takashi Mori, Hiroyuki Tomita, Takayuki Mori, Hirohiko Imai, Masayuki Matsuo

**Affiliations:** 1 Department of Radiology, Graduate School of Medicine, Gifu University, Gifu, Gifu, Japan; 2 Department of Radiology, Asahi University Hospital, Gifu, Gifu, Japan; 3 Department of Pharmacology, Graduate School of Medicine, Gifu University, 1-1 Yanagido, Gifu, 501-1194 Japan; 4 Center for One Medicine Innovative Translational Research (COMIT), Institute for Advanced Study, Gifu University, Gifu, Japan; 5 Innovation Research Center for Quantum Medicine, Graduate School of Medicine, Gifu University, Gifu, Japan; 6 Department of Frontier Science for Imaging, Gifu University, Gifu, Japan; 7 Department of Food Hygiene and Control, Faculty of Veterinary Medicine, Suez Canal University, Ismailia, Egypt; 8 Department of Tumor Pathology, Gifu University Graduate School of Medicine, Gifu, Japan; 9 Joint Department of Veterinary Medicine, Faculty of Applied Biological Sciences, Gifu University, Gifu, Japan

**Keywords:** DNP-MRI, Redox, Imaging, Radiotherapy, Nitroxyl radicals, Intestines

## Abstract

The intestine is an important organ in radiotherapy of the abdominal region, and radiation-induced intestinal injury (RIII) is an undesirable biological response to radiotherapy. Radiotherapy is known to induce oxidative stress associated with the generation of reactive oxygen species (ROS), which in turn plays an important role in RIII. However, these effects cannot be detected or predicted early using conventional imaging techniques such as computed tomography and magnetic resonance imaging (MRI). In this study, an intestinal redox imaging method using dynamic nuclear polarization (DNP) MRI and carbamoyl PROXYL (CmP) was designed. The probe was prepared in a solution of increased viscosity of the CmP solution that is not affected by peristalsis. This redox imaging method enabled noninvasive redox imaging of the intestine and detection of RIII at an early stage of progression. Our findings suggest that redox imaging can aid in monitoring early metabolic changes that occur during the pathogenesis of this condition.

**Supplementary Information:**

The online version contains supplementary material available at 10.1186/s11671-025-04355-y.

## Introduction

The intestine is an important organ in radiotherapy of the abdominal region; moreover, it is a radiosensitive organ compared with other organs. In particular, the mucosal epithelial cells of the intestine are sensitive to irradiation [1, 2]. Intestinal perforation also may occur when the intestines are exposed to excessive radiation, resulting in a life-threatening condition called radiation-induced intestinal injury (RIII). The therapeutic and adverse effects of radiotherapy are basically assessed based on morphological changes observed using conventional imaging modalities, such as computed tomography and magnetic resonance imaging (MRI); tumor and normal tissue findings obtained using contrast media; radioactive imaging techniques, such as ^18^F-fluorodeoxyglucose-positron emission tomography; and blood tests [3–5]. However, it is difficult to determine the efficacy of treatment and adverse inflammatory complications at the exposure site at an early stage. Inflammatory changes in tissues are known to appear early, and radiation has been shown to induce oxidative stress in the living tissues, causing acute and chronic cellular damage; this damage in the gut is considered important during radiotherapy [6]. For this reason, the diagnosis of inflammatory reactions may allow the initiation of proactive measures and preliminary treatment at an early stage. Therefore, a technique that can detect changes in tissue conditions early is required.

The effects of irradiation on living tissues are divided into direct and indirect effects. In the direct effects, when radiation passes through a living cell, it interacts with its biomolecules, such as deoxyribonucleic acid (DNA), causing ionization and excitation of the biomolecules, interrupting the biochemical reactions, and resulting in biological changes. In the indirect effects, ionization and excitation of the cellular water molecules occur, and reactive oxygen species (ROS) are generated. These produced ROS, such as hydroxyl radicals, oxidize the nearby DNA, protein molecules, cell membranes, etc. and disrupt the redox balance, which eventually leads to cell death and various adverse biological effects [7–9].

**Fig. 1:**
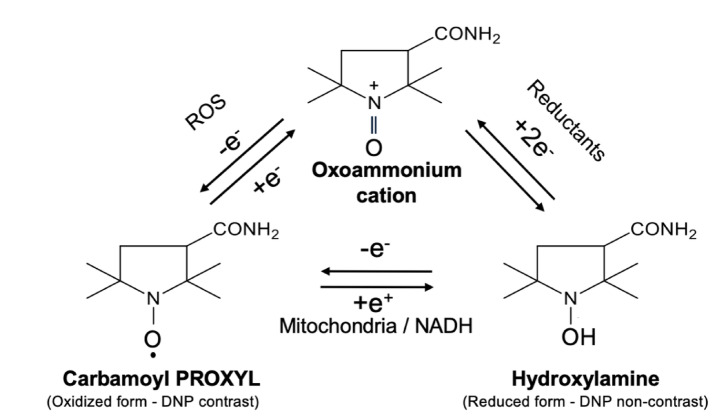
Mechanisms of CmP redox and DNP contrast. CmP converts to hydroxylamine or oxoammonium cation in vivo. CmP is found in vivo in an equilibrium between the nitroxide radical form, which is detectable by EPR, and the reduced hydroxylamine form, which is not detectable

Because the ROS and other free radicals generated in living organisms occur in minute frame and disappear within a short time, their direct measurement has long been considered challenging. Therefore, redox experiments have been conducted using nitroxyl radicals, which are relatively stable compounds, as probes to detect redox reactions [10, 11]. One of these radicals, 3-carbamoyl-2,2,5,5-tetramethyl-3-pyrrolidine-1-oxyl (carbamoyl PROXYL; CmP) [12], is a stable low-molecular-weight compound that has been shown to have low biotoxicity [13]. The biological reaction mechanism of CmP involves its oxidation by ROS to form oxoammonium cations, which are further reduced by reducing molecules such as glutathione in the body to produce hydroxylamine (Fig. 1) [12]. Thus, the reduction rate of CmP can acutely reflect the redox reaction of tissues [14]. Furthermore, CmP is also used as a contrast agent in dynamic nuclear polarization (DNP) MRI systems because it has radicals in its structures and these radicals can be polarized in vivo [14, 15].

In vivo DNP-MRI, also called Overhauser-enhanced MRI [16, 17] or proton-electron double resonance imaging [18, 19], is a technique used to assess the biological reactions in vivo. In this technique, a stable free radical compound, such as CmP, is injected in vivo to create a situation in which electron and proton spins are mixed, and then the free radical molecule is irradiated with electromagnetic waves at a frequency that causes electron spin resonance (ESR) to induce DNP in vivo [17, 19–23]. Yasukawa et al. reported redox imaging of dextran sodium sulfate-induced colitis in mice with minimally invasive methods using ^15^N-labeled CmP, and the production of ROS was found to be related to colitis [24, 25]. For noninvasive imaging of the intestines, it may be important to allow the DNP probe to remain in the intestinal tract for a while. Therefore, in this study, the CmP was mixed with hyaluronic acid (HA) to increase its viscosity and stability against peristalsis [26]. Furthermore, we succeeded in monitoring the early redox alterations of the intestines after total-body irradiation (TBI).

## Material and methods

### Chemicals

CmP was purchased from Sigma-Aldrich Chemical Co. (Milwaukee, WI, USA). HA was purchased from FUJIFILM Wako Pure Chemical Co (Osaka, Japan). All other chemicals were of reagent-grade quality and were obtained from commercial sources.

### Phantom study: CmP/HA solution

**Fig. 2:**
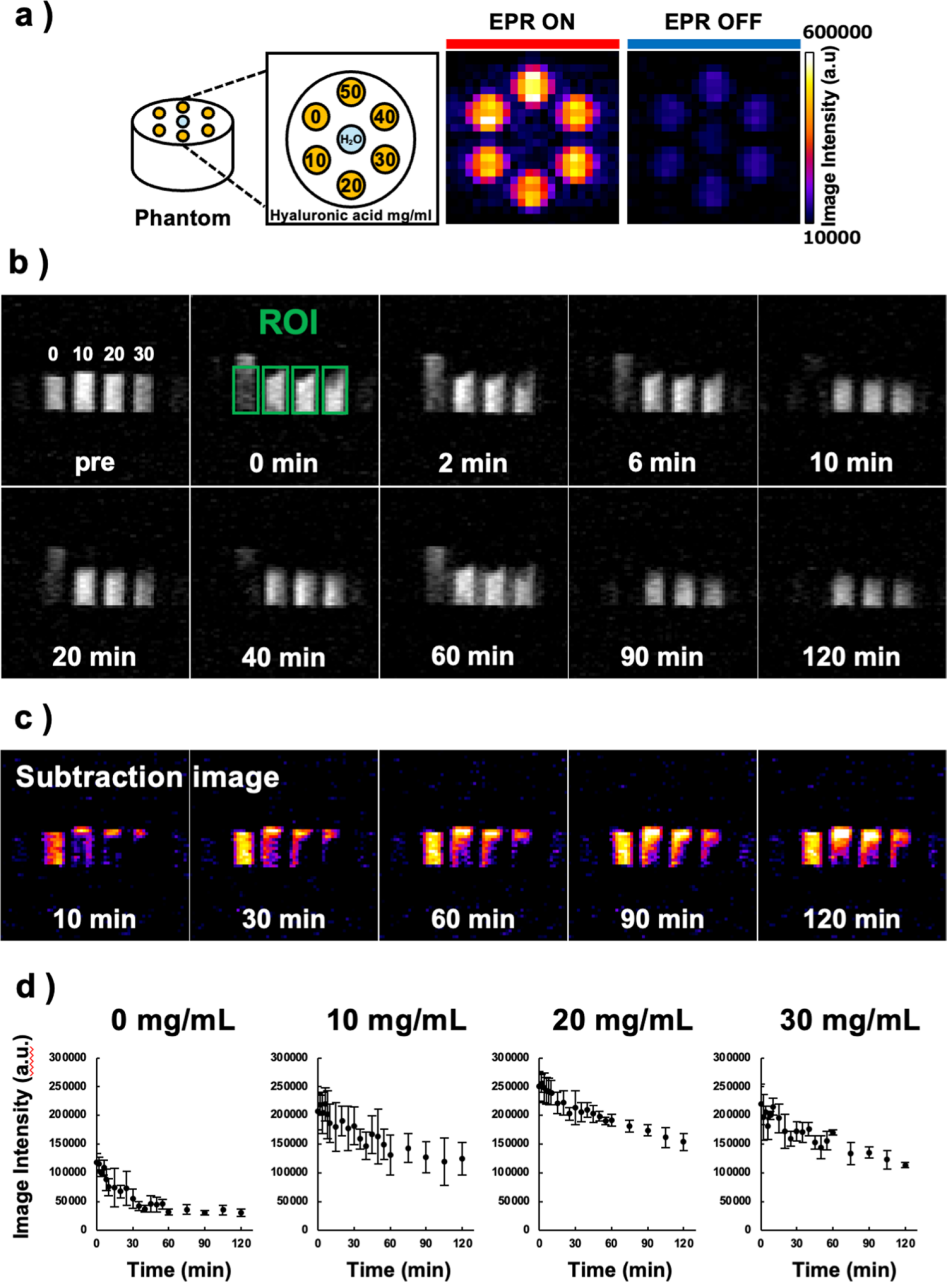
DNP-MR imaging of CmP/HA phantom. **a** Schematic and DNP image of CmP/HA phantom (CmP: 2 mM) with different concentrations of HA (0, 10, 20, 30, 40, and 50 mg/mL.) **b** Pharmacokinetic DNP-MR images of CmP/HA solution with AsA. **c** Subtracted images of pharmacokinetic DNP-MR images. Subtracted images were generated by subtracting the images acquired at each time point (10, 30, 60, and 90 min) from the prereaction image. **d** Profile curves of DNP images. Curves were created from the image intensities of the DNP images. Data are expressed as mean ± standard deviation (n = 3, each sample)

Various concentrations of HA (10, 20, 30, 40, and 50 mg/mL) were tested in mixtures with CmP. The central well of the phantom was filled with H_2_O, and all six surrounding wells were filled with the CmP/HA solution (Fig. 2a). Imaging was conducted using a low-field DNP-MRI system (Keller) obtained from Japan Redox Ltd. (Fukuoka, Japan). The external magnetic field B_0_ for electron paramagnetic resonance (EPR) irradiation and MRI was fixed at 15 mT, and the radiofrequencies of the EPR irradiation and MRI were 458 MHz and 689 kHz, respectively. A whole-body coil for EPR irradiation was used for DNP-MRI imaging. The scanning conditions for the DNP-MRI experiment were as follows: power of EPR irradiation, 5 W; flip angle, 90°; repetition time (TR) × echo time (TE) × EPR irradiation time (TEPR), 1000 × 20 × 500 ms; accumulation number, 10; slice thickness, 30 mm; phase-encoding steps, 32; field of view (FOV), 50 × 50 mm; and matrix size, 64 × 64 after reconstruction. DNP-MRI images were obtained with EPR irradiation at the time of imaging. Next, normal MRI images were taken without EPR irradiation and all images were then analyzed using ImageJ software (version 1.54g).

The redox reaction of CmP was monitored by mixing 0, 10, 20, and 30 mg/mL HA with 2 mM CmP and adding these mixtures to four test tubes. Then, 200 µL of 20 mM ascorbic acid (AsA) was added to each tube and imaging was performed (pre, 0, 2, 6, 10, 20, 40, 60, 90, and 120 min). The scanning conditions were the same as those in the earlier phantom study; however, the slice thickness was set to 100 mm (covered whole test tubes thickness) and DNP-MRI images were taken with EPR irradiation at the time of imaging. The DNP-MRI images were processed and analyzed using ImageJ software, and changes in the signal intensity were measured over time.

### Animals

C57BL/6 mice, aged 6 weeks, were purchased from Charles River Laboratories, Inc. (Yokohama, Japan) and housed in a climate-controlled room with a 12:12-h light/dark cycle. The mice were allowed free access to water and food (Moderate Fat diet, Oriental Yeast Co., Tokyo, Japan) and acclimatized to their environment for 1 week before the experimental process began. All animal care and experimental procedures were approved by the Gifu University Animal Experiment Committee and conducted following the ethics of the Gifu University Animal Experiment Committee. The mice were fasted (with free access to drinking water) during DNP-MRI imaging. Every effort was made to minimize the number of mice used and their suffering.

### Imaging and macroscopic examination of the localization of the redox probe

To test the retention of the redox probe in the intestine, CmP solution (2 mM) and CmP/HA (2 mM CmP, 30 mg/mL HA) solution mixed with Evans Blue stain [27, 28] were prepared for in vivo redox imaging of the lower abdomen. Nonirradiated mice were anesthetized by isoflurane inhalation (4% in the induction phase and 1.5% in the maintenance phase) in medical air (400 mL/min). In vivo DNP-MRI imaging was started after rectal administration of the CmP or CmP/HA solution to the mice. The scanning conditions for the DNP-MRI experiment were as follows: power of EPR irradiation, 5 W; flip angle 90°; TR × TE × TEPR, 500 × 37 × 500 ms; accumulation number, 1; slice thickness, 100 mm; phase-encoding steps, 32; FOV, 60 × 60 mm; and matrix size, 64 × 64 after reconstruction. In vivo DNP-MRI images were obtained at 1, 3, 5, 7, 9, 11, 13, and 15 min after administration of the CmP or CmP/HA solution. After imaging, pharmacokinetic images (DNP images) were created, and the regions of interest (ROIs) of the colon were analyzed using ImageJ software. The in vivo reduction rates were then calculated from the decay of the DNP enhancement in each image pixel using Microsoft Excel software. The mice were then sacrificed, the abdominal regions were dissected, and the intestines were extracted and visually examined.

### X-ray irradiation

Using a linear accelerator (Primus Mid-Energy 4 MV linear accelerator; Siemens Healthcare, Malvern, PA, USA) at Gifu University Veterinary Hospital, all mice were exposed to a single dose of TBI at a dose of 10 Gy X-rays under nonanesthetic conditions. The irradiated mice (TBI group) were then divided according to examination time point after irradiation into four groups: 1 h, 1 day, 3 days, and 1 week (five mice in each group).

### Monitoring the intestinal redox status by in vivo DNP-MRI

Four TBI groups and a control group of nonirradiated mice (five mice per group) were tested by in vivo DNP-MRI for their intestinal redox status. Mice were first anesthetized by isoflurane inhalation (4% for induction and 1.5% for maintaining anesthesia) in medical air (400 mL/min). In vivo DNP-MRI scans of the intestines were then performed in each mouse following rectal administration of CmP/HA solution (2 mM CmP, 30 mg/mL HA). The scanning conditions for the DNP-MRI experiment were as follows: power of EPR irradiation, 5 W; flip angle, 90°; TR × TE × TEPR, 500 × 37 × 500 ms; accumulation number, 1; slice thickness, 100 mm; phase-encoding steps, 32; FOV, 60 × 60 mm; and matrix size, 64 × 64 after reconstruction. The in vivo DNP-MRI images were taken at 1, 3, 5, 7, 9, 11, 13, and 15 min after administration of the CmP/HA solution. Subsequently, conventional MRI anatomical images with 10 accumulations without EPR irradiation were obtained. After imaging, the pharmacokinetic images (DNP images) from the ROIs of the intestines were created using ImageJ software, and the in vivo redox change was then calculated from the decay slope of the signal intensity of the enhanced DNP image in each image pixel using Microsoft Excel software.

### EPR spectroscopic analysis of the redox reaction of CmP

We used a JEOL RESONANCE JES-X310 X-band ESR spectrometer (JEOL Ltd. Tokyo, Japan) in the ESR spectrometry experiments. One nonirradiated group (five mice) and four TBI groups (five mice in each group) were used for these experiments. Mice were sacrificed after deep anesthesia using 4% isoflurane. The intestines were extracted and then mixed with the mitochondrial assay solution, and a homogenate solution was prepared by grinding the intestines in a homogenizer device while chilling them in ice water. Then, the tissue homogenates were heated in 37 °C water for 1 min, mixed with CmP, CmP + β-nicotinamide adenine dinucleotide hydrate (NADH), or CmP + NADH + potassium cyanide (KCN), and subjected to ESR measurement. ESR spectra were measured at 1, 3, 5, 7, 9, 11, and 13 min after reagent addition. The measurement conditions for the ESR spectrometer experiment were as follows: microwave frequency = 9.4 GHz (336 mT); microwave power = 10 mW; modulation width = 0.6 mT; sweep time = 1 min; sweep width =  ± 5 mT; and time constant = 0.03 s.

### Histopathology

One nonirradiated (control) and four TBI groups of mice were examined. Mice were deeply anesthetized with 4% isoflurane, sacrificed, and subjected to intestinal extraction. The extracted intestinal samples were fixed in 10% formalin neutral buffer solution and embedded in paraffin blocks. The blocks were cut into 3-µm sections, stained with hematoxylin and eosin or Masson’s trichrome and observed using a microscope (BZ-X 800, KEYENCE, Osaka, Japan). These tissues were evaluated for the number of goblet cells per crypt [29], neutrophils infiltrating the intestinal epithelial cells in a random FOV, and lymphocytes [30] as pathological findings of radiation injury.

### Statistical and analysis

Differences in group means were determined using Student’s* t*-test, and significance thresholds were set at *p* < 0.05 and *p* < 0.01.

## Results

Figure 2a shows the DNP image of the phantom containing H_2_O and various concentrations of HA with 2 mM CmP. All phantom wells of CmP/HA were clearly enhanced by DNP and showed similar DNP image intensities. When the EPR irradiation was turned off, all wells containing CmP/HA solution and H_2_O showed no DNP enhancement. In addition, no change in EPR signal intensity was observed in CmP solutions with varying concentrations of HA, as shown in Supplementary Fig. 1. Figure 2b shows the results of DNP-MRI before and after the addition of AsA solution for the evaluation of the redox reaction with the CmP/HA mixture. After AsA was added, the redox reaction started, and the DNP image intensity decreased over time. The reduction of DNP enhancement and region differed based on the HA concentration. Although the tube without HA showed a clear reduction in the DNP image intensity in the whole tube, the HA-containing tubes showed a reduction mainly in the upper areas of the probe mixtures (Fig. 2c). The profile curves of the DNP image intensity showed that CmP without HA had the fastest decay rate, followed by that of the 10 mg/mL of HA (Fig. 2d). While HA possesses antioxidant activity, it was used in this study to primarily increase viscosity and retention time after rectal administration. Due to its high molecular weight, HA has very low cell membrane permeability. In contrast, CmP can penetrate the cell membrane. Our phantom experiments with AsA confirmed that CmP retained redox activity even in the presence of HA (Fig. 2). These results suggest that CmP was able to permeate intestinal tissue and mediate redox reactions after administration, enabling efficient detection of redox changes in irradiated tissues via DNP-MRI. Therefore, we used 30 mg/mL HA in this study as it was expected to improve probe retention.

**Fig. 3:**
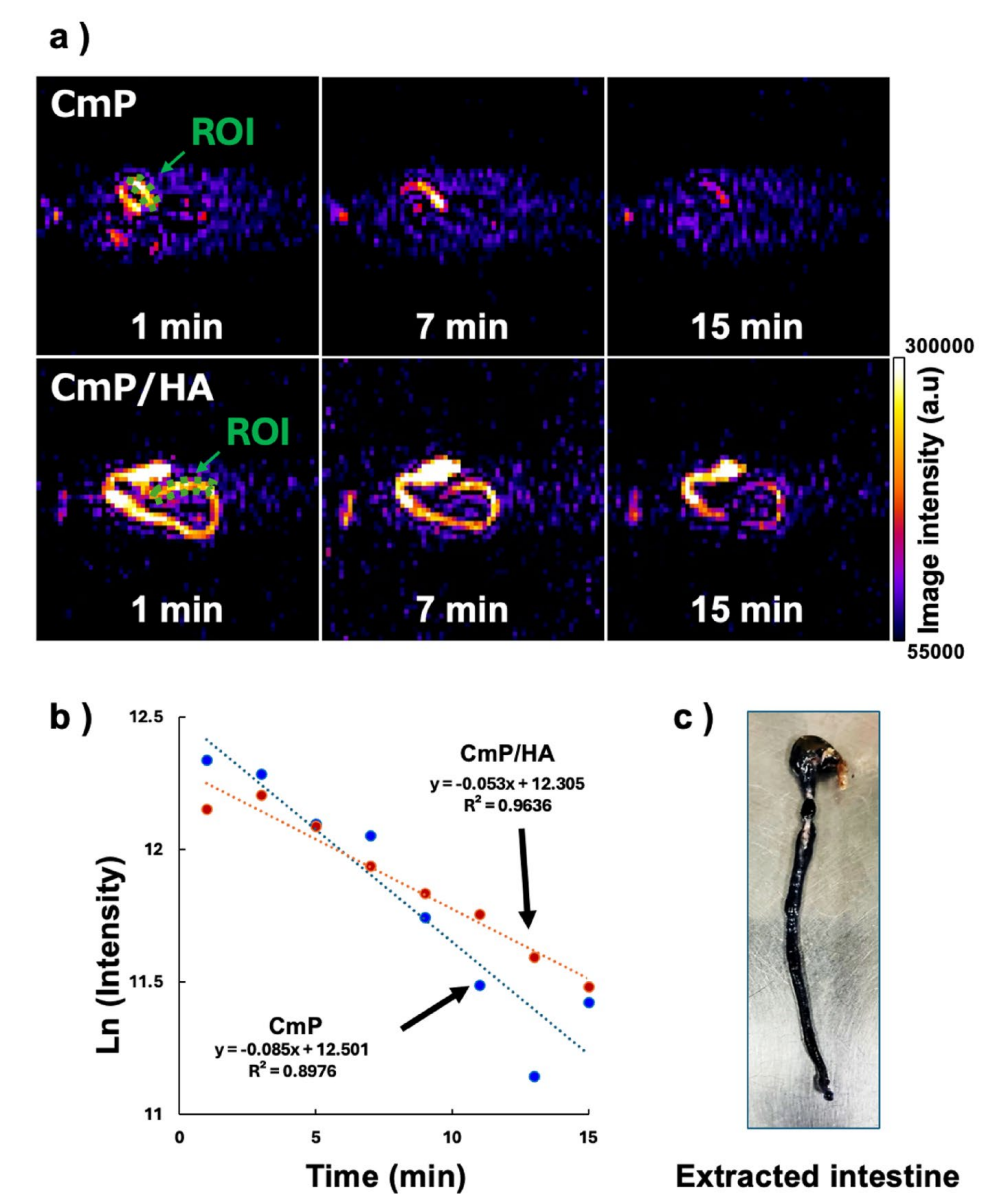
Evaluation of the in vivo redox imaging of the intestine using DNP-MRI and CmP/HA probe. **a** Representative DNP-MR images visualizing the pharmacokinetics of CmP and CmP/HA. **b** Decay curves of CmP based on the DNP-MR images. ROIs were created in the intestinal tract based on the CmP kinetic images. CmP decay curves and their linearity were calculated from the slope of each pixel. **c** Picture of isolated intestine after DNP-MR imaging

To evaluate the retention of the CmP/HA in the intestinal tract, control mice were administered CmP or CmP/HA solutions, and in vivo DNP-MRI was conducted (Fig. 3a). In mice administered with both the CmP and CmP/HA solutions, the distribution of CmP in the lower abdominal region was visualized. In particular, when the CmP/HA was used, the intestinal structures were clearly visualized for a longer time. The decay rate of CmP, calculated from the DNP image intensities, decreased steadily over time in both cases (Fig. 3b). When CmP alone was used, a low linearity of the regression curve (*R*^2^ = 0.898) and improper distribution of CmP along the measurement time were observed. In contrast, the CmP/HA solution showed higher linearity of the regression curve (*R*^2^ = 0.964) and better CmP distribution. Isolation of the intestines from the abdomen of mice after DNP-MRI showed that the CmP/HA solution was retained in the intestine despite the decrease in the amount of radicals (Fig. 3c).

**Fig. 4:**
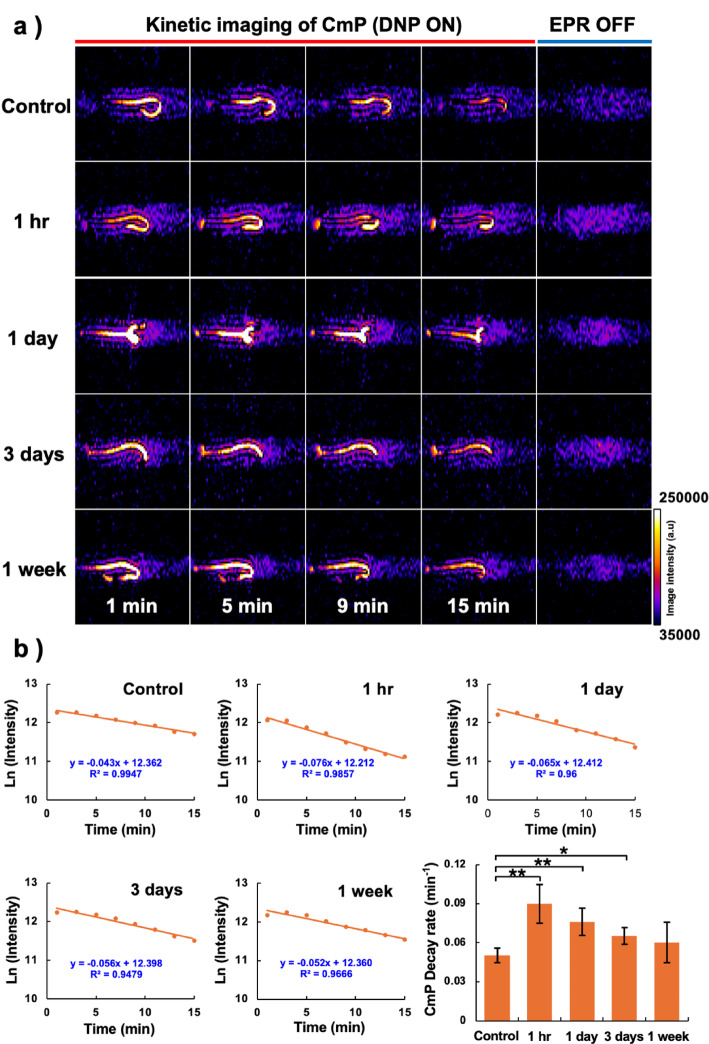
In vivo DNP-MRI monitoring of CmP/HA after TBI. **a** In vivo pharmacokinetic DNP-MRI images of CmP after rectal administration of CmP/HA **b** decay curves and rates of CmP. The ROI was created in the intestinal tract based on the CmP kinetic images, and the in vivo CmP decay curves and rates were calculated from the slope of each pixel. Data are expressed as the mean ± standard deviation (n = 5, each group). *p < 0.05, **p < 0.01

In vivo DNP-MRI clearly visualized the distribution of CmP in the lower intestinal region of the nonirradiated (control) and TBI mice groups (Fig. 4a). In addition, the image intensities of the intestinal tract gradually decreased in all groups with time. Figure 4b shows the decay curves and rates of CmP in all groups. At 1 h post-irradiation, the CmP decay rate was the fastest (**p < 0.01), and it decreased gradually. In addition, the CmP distributed in the intestinal region was successfully visualized as a clear three-dimensional (3D) image, as shown in Supplementary Fig. 2.

**Fig. 5:**
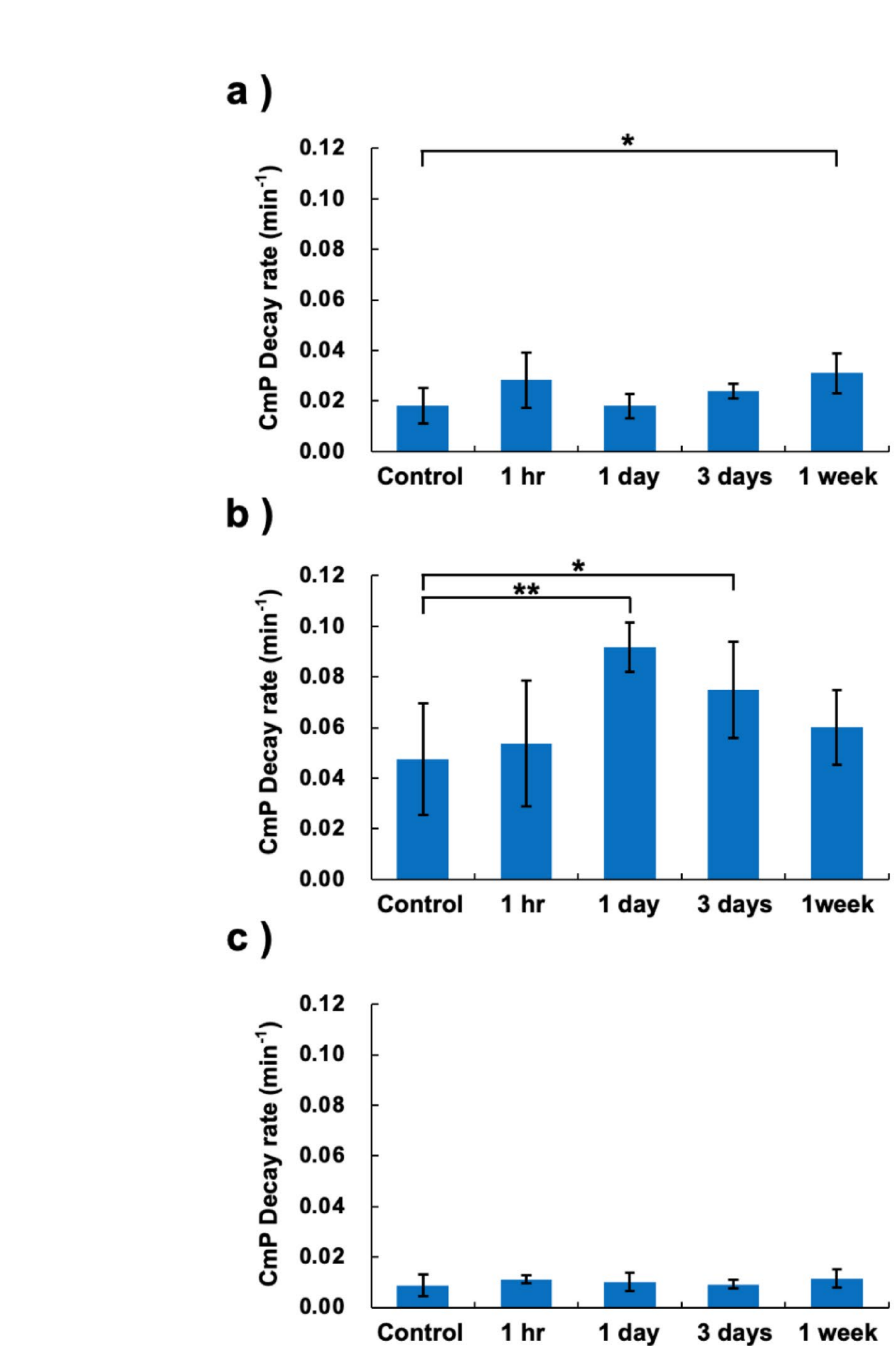
Ex vivo redox monitoring of the intestine using EPR. Data are expressed as mean ± standard deviation (n = 5, each group). *p < 0.05, **p < 0.01. **a** The decay rate of CmP in the intestine of the control and TBI mice groups. **b** The decay rate of the CmP with NADH. **c** The decay rate of CmP with NADH and KCN

The ESR analysis confirmed that the intestinal tissue homogenate had redox reactivity with CmP (Fig. 5). The TBI groups showed different redox reaction rates at each elapsed time after irradiation. In the CmP group (Fig. 5a), the reduction of CmP was enhanced 1 h post-irradiation, decreased on day 1, and recovered again 1 week post-irradiation (*p* < 0.05). The decay rate of CmP was increased in samples treated with NADH, a substrate of the mitochondrial electron transfer chain (ETC) (Fig. 5b). The CmP decay rate increased the most after 1 day of irradiation and then gradually decreased. In the samples treated with KCN, a mitochondrial ETC inhibitor, there was a suppletion of the decay rate that was much slower than that of the other samples, and there were no significant differences between the control mice samples and those of the irradiated mice (Fig. 5c).

**Fig. 6:**
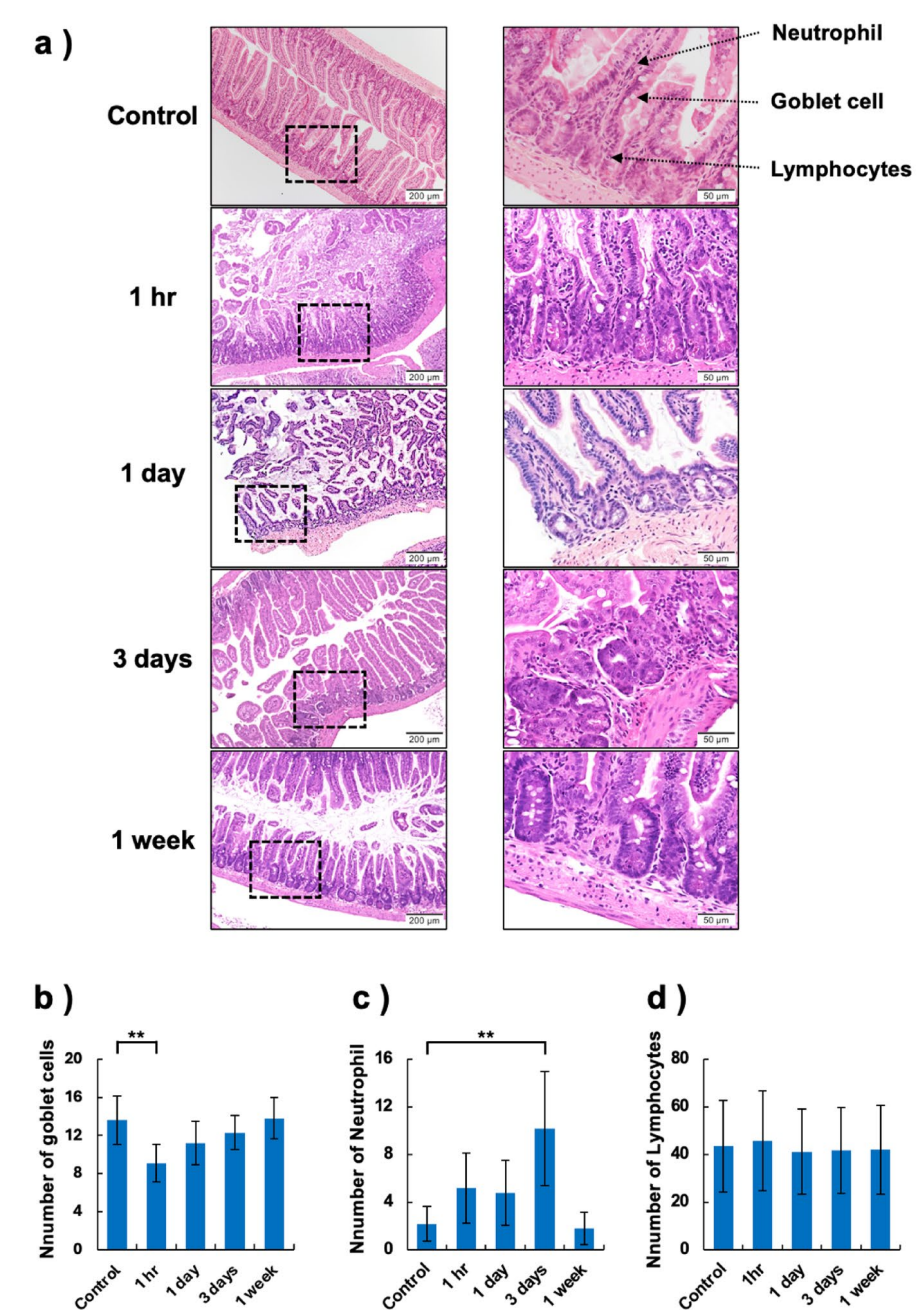
Histopathological evaluation of intestinal tissue damage. **a** Histopathological images of the intestine obtained from nonirradiated and TBI mice. The lower images are at a larger scale. **b** Number of goblet cells per crypt. **c** Number of neutrophils infiltrating the intestinal epithelial cells in a random field of view. **d** Number of lymphocytes infiltrating the intestinal epithelial cells in a random field of view

Figure 6a shows the results of the histopathological evaluation of the intestines in control and TBI mice groups. The number of goblet cells per crypt was lowest at 1 h post-irradiation (**p < 0.01) (Fig. 6b). Regenerative changes were observed from 3 days post-irradiation. The number of goblet cells was close to that of the control at 1 week. A greater number of neutrophils infiltrating the intestinal epithelial cells was observed at 1 h post-irradiation. Moreover, a significant increase in neutrophils was observed 3 days (**p < 0.01) post-irradiation (Fig. 6c). There was no significant difference in the number of lymphocytes infiltrating the intestinal epithelial cells (Fig. 6d).

## Discussion

When HA was mixed with CmP to increase retention in the intestine, the intensity of the DNP-MRI image did not change significantly compared with that in the CmP-only case. The redox reaction of CmP was also detectable when HA was added to the probe solution (Fig. 2c) [14]. In addition, no differences in the ESR signal intensities of CmP were observed as a result of HA mixing (Supplementary Fig. 1). This unchanged ESR spectrum suggests that the CmP radical retained redox reactivity. These results indicate that the addition of HA to the CmP solution does not affect redox imaging using in vivo DNP-MRI. Therefore, we considered a highly viscous HA concentration of 30 mg/mL to be the optimal concentration for measuring stable redox reactions. In vivo DNP-MRI clearly visualized the distribution of CmP in the intestine after rectal administration and redox imaging of the intestine after radiation treatment was successfully detected at an early stage. Furthermore, the 3D image construction made it easier to understand the structure of the intestinal tract. The image intensity of the intestine in each group decreased with time, indicating that the redox reaction of the CmP/HA solution occurs based on the redox status of the intestinal tract [31, 32]. Furthermore, the administration of the colored CmP/HA solution showed that the CmP/HA solution clearly remained in the isolated intestinal tract even when the CmP radicals were completely reduced (Fig. 3c). Therefore, the observed alteration of the intensity of the DNP image does not indicate CmP clearance from the intestinal tract but rather its reduction based on the redox reaction. These findings suggest that the CmP/HA solution might be a suitable probe for the noninvasive evaluation of the intestinal redox response. The TBI groups showed differences in the metabolic rates of CmP at all time points after irradiation, and these differences were statistically significant (**p < 0.01) at 1 h post-irradiation. Garg et al. [33] reported that a single 8-Gy γ-ray dose to mice decreased the number of neutrophils, macrophages, B lymphocytes, and T lymphocytes at 4 h post-irradiation. Jameus et al. [34] reported that the number of goblet cells in the intestine decreased as early as 48 h post- irradiation with 0.1–3-Gy X-rays in the same mouse strain. Similarly, Yuan et al. [35] demonstrated that DNA damage, indicated by phosphorylated histone H2AX, decreased within 24 h following 12-Gy TBI and then began transitioning to a repair phase. These findings support the idea that redox alterations may occur early after irradiation, reflecting a complex combination of cell damage and adaptive repair responses. Because we used a single and relatively high dose irradiation (10-Gy X-rays), we expected that changes in redox balance might occur within a shorter time frame. Therefore, we could probably detect RIII early in the intestinal redox imaging.

The redox reaction of the CmP solution with the intestinal tissues was verified by an ex vivo study using isolated intestinal samples. When tissue homogenates treated with CmP alone and CmP + NADH were pretested using ESR, the decay rate of CmP + NADH was increased in all groups. This is because NADH is a substrate of the mitochondrial ETC for adenosine triphosphate synthesis [36]. Conversely, the reduction of CmP was suppressed when KCN was added. This suggests that the mitochondrial redox metabolism is strongly involved in the reduction of CmP [37]. In our comparison of the NADH- and KCN-exposed tissues, we observed the maximum increase in mitochondrial metabolism 1 day post-irradiation. Radiation-induced ROS generation, coupled with the intricate interplay of activated intracellular mechanisms, mitochondrial dysfunction, and impaired antioxidant defenses, is known to lead to the spread of oxidative stress throughout the cell tissue [38]. This also suggested that the increase in metabolism 1 h post-irradiation might be attributed to the activation of not only mitochondria but also the entire cellular tissue.

When intestinal stem cells (ISCs) function normally, they proliferate rapidly to regenerate multiple adjacent crypts and villi after injury [39]. However, if ISCs suffer fatal damage, the lost cells cannot be replenished in a short period, leading to a breakdown of intestinal integrity. Conversely, a decrease in the proliferation activity of ISCs results in villus shortening and impaired intestinal function. Thus, inflammatory changes in intestinal epithelial cells have been reported to decrease the number of goblet cells and the infiltration of neutrophils and lymphocytes into the intestinal epithelium [33–35, 40–42]. 1 h post-irradiation, the number of goblet cells per crypt was the lowest. This early dropout of goblet cells might have occurred because the extent of the critical damage was greater than that reported by Garg et al. and Jameus et al. [33, 34]. In addition, the number of neutrophils in the intestinal epithelial cells also increased 1 h post-irradiation. The results of the ESR and in vivo DNP-MRI also showed that the redox reaction was most enhanced 1 h post-irradiation, suggesting increased ROS in intestinal tissues. Pathological findings showed that changes in goblet cell regeneration due to tissue damage were observed at 3 days post-irradiation, and at the same time, neutrophil infiltration of intestinal epithelial cells (**p < 0.01) showed the greatest increase, suggesting a progression in tissue damage. Subsequently, the increase in goblet cells observed at 1 week post-irradiation may indicate increased mucus secretion to protect intestinal epithelial cells from tissue damage.

These results demonstrate that the enhancement of the redox response throughout the cellular tissue due to radiation damage began during the 1 h post-irradiation. Therefore, in vivo DNP-MRI may reflect RIII at an early stage before the condition becomes complicated using the CmP/HA solution.

## Summary

In this study, we developed a noninvasive and stable in vivo DNP-MRI method for intestinal redox imaging using a high-viscosity HA-based redox probe to enhance the retention of CmP. This technique demonstrated potential for the early detection of tissue damage after radiation treatment and may serve as a valuable biomarker for early monitoring of RIII.

## Supplementary Information

Below is the link to the electronic supplementary material.

Supplementary Material 1

## Data Availability

The data will be made available on reasonable request.

## Declarations

### Clinical trial number

Not applicable as this study involved animal subjects and did not include human participants.
