# Supplementary material for: Development of a noninvasive redox imaging method that can stably detect radiation-induced intestinal injury
Source: Discov Nano. 2025 Sep 24;20(1):166. doi: 10.1186/s11671-025-04355-y (PMC12460868; doi:10.1186/s11671-025-04355-y)
Supplement: Supplementary file 1 — Supplementary Material 1 [file 11671_2025_4355_MOESM1_ESM.docx]

**Supplementary information**

**Supplementary materials and method**

**Study of changes in the spectral intensity of the CmP/HA solution**

For the EPR analysis, a JEOL RESONANCE JES-X310 Spectrometer was used. HA was mixed with the CmP solution (2 mM) at 0, 10, 20, 30, 40, or 50 mg/mL. The signal intensities were compared and verified by ESR measurement. The measurement conditions were as follows: microwave frequency = 9.4 GHz (336 mT); microwave power = 10 mW; modulation width = 0.6 mT; sweep time = 1 minute; sweep width = ±5 mT; and time constant = 0.03 seconds.

**3D DNP-MRI of the CmP/HA solution distribution**

*In vivo* 3D imaging was conducted using a low-field DNP-MRI system (Keller) obtained from Redox Japan (Fukuoka, Japan). During imaging, the mice were anesthetized by isoflurane inhalation (4% for anesthesia induction and 1.5% for anesthesia maintenance) in medical air (400 mL/min). *In vivo* DNP-MRI scans of the intestinal tract were performed after rectal administration of CmP/HA solution (2 mM CmP, 30 mg/mL HA). *In vivo* DNP-MRI images were obtained 6 and 11 minutes after the EPR irradiation at the time of imaging. Volume rendering images with a slab depth of 30 mm were created using the INTAGE Realia software. The scan conditions for the DNP-MRI experiment were as follows: power of EPR irradiation, 5 W; flip angle 90°; TR × TE × TEPR, 500 × 37 × 500 ms; accumulation number, 1; slice thickness 100 mm; slab phase, 32; phase-encoding steps, 32; FOV, 60 × 60 mm; and matrix size, 64 × 64 after reconstruction.

**Supplementary results**

Spectral measurements were conducted to confirm that the addition of HA did not affect the signal of CmP (Supplementary Figure 1). No significant difference in the spectral signal was observed, even with increased viscosity.


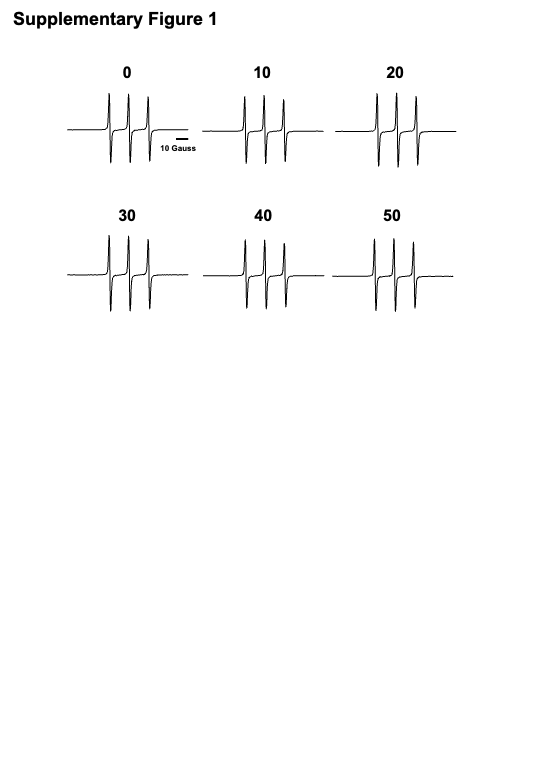


Furthermore, the stagnation of the CmP/HA solution in the intestine was clearly visualized, enabling the successful reconstruction of a 3D image of the intestinal structure (Supplementary Figure 2).


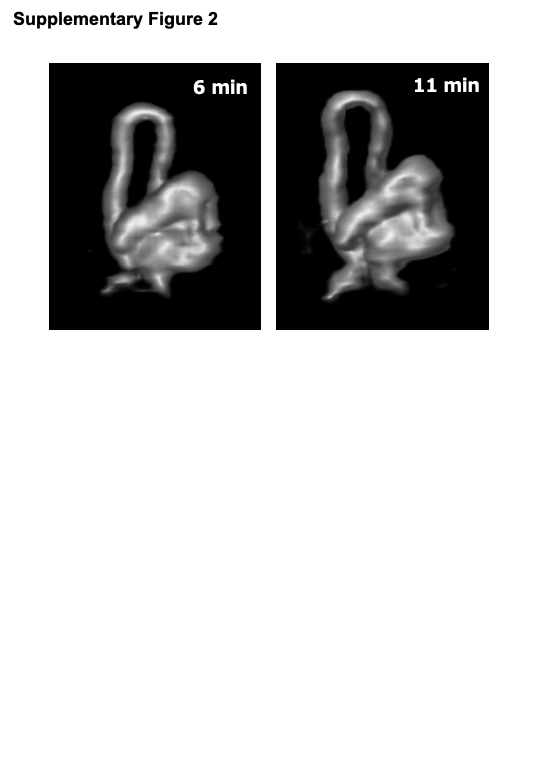


**Supplementary discussion**

The unchanged ESR spectrum suggests that the CmP radical retains a stable structure and does not bind to other molecules, including HA. The 3D visualization of CmP/HA distribution facilitates a clearer understanding of the intestinal tract’s structural features.
